# Synthesis of Some Novel Pyrazolo[1,5-a]pyrimidine Derivatives and Their Application as Disperse Dyes

**DOI:** 10.3390/molecules16065182

**Published:** 2011-06-22

**Authors:** Alya M. Al-Etaibi, Nouria A. Al-Awadi, Morsy A. El-Apasery, Maher R. Ibrahim

**Affiliations:** 1 Natural Science Department, College of Health Science, Public Authority for Applied Education and Training, Kuwait; 2 Chemistry Department, Faculty of Science, Kuwait University, P.O. Box 5969, Safat 13060, Kuwait

**Keywords:** enaminone, pyrazolohydrazone, microwave irradiation, disperse dyes, HMBC 2-D, 2,4-pentandione, enaminonitrile

## Abstract

A series of novel monoazo-disperse dyes containing pyrazolo[1,5-a]pyrimidine structures were synthesized starting with the coupling reaction between ethyl cyanoacetate and 4-hydroxybenzenediazonium chloride, followed by treatment of the resulting hydrazone product with hydrazine hydrate. The pyrazolohydrazone **6** is then treated with either 2,4-pentandione and enaminonitrile or aryl-substituted enaminoketones to give the target pyrazolo[1,5-a]pyrimidine dyes **7** and **15a-d**. Structural assignments to the dyes were made using NMR spectroscopic methods. A new high temperature method, using microwave heating, was employed to apply these dyes to polyester fibers. Most of the dyed fabrics tested displayed moderate light fastness and excellent washing fastness properties.

## 1. Introduction

Pyrazole derivatives are important intermediates in organic synthesis and possess a range of interesting biological and antimicrobial properties [1,2,3,4,5,6,7,8]. Their fused pyrimidine derivatives are used as dyes [9,10,11,12,13,14]. In the current study, we prepared new azopyrazolopyrimidine dyes, starting with 3-amino-4-(4-hydroxyphenyl)azo-1H-2-pyrazoline-5-ones and 3-(4-hydroxyphenylazo-1H-2-pyrazoline-5-ones, and applied them to polyester fibers as disperse dyes by using a new high temperature microwave heating method. Microwave irradiation leads to a large increase in dye uptake and dyeing rate, along with a performance of dye leveling and color homogeneity than can be achieved by using conductive heating [15,16,17]. 

## 2. Results and Discussion

### 2.1. Synthesis and Characteristics

**Scheme 1:**
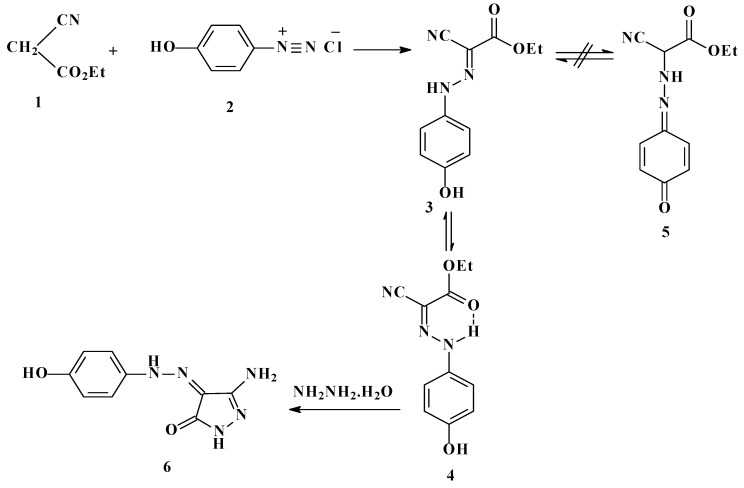
Preparation of hydroxyphenylhydrazonopyrazolone **6**.

Ethyl 2-arylhydrazonocyanoacetate **4 **was formed by addition under mild conditions of ethyl cyanoacetate (**1**) to 4-hydroxybenzenediazonium chloride (**2**) that readily affords the corresponding hydrazone **3**. The existence of this substance in the quininoid form **5 **was ruled out based on the results of NOE difference experiments, which showed that irradiation of the two NH signals at 12.1 and 13.0 ppm caused an enhancement of the intensities of the aryl proton resonances at 6.3 and 6.8 ppm, while irradiation of the two OH signals at 9.68 and 9.57 ppm also resulted in an increase in the intensities of the aryl proton peak at 6.8 ppm. Moreover, the ^13^C-NMR spectrum of **3 **shows only two sets of two sp^3^ carbon signals at 61.7 and 61.2, and 14.20 and 14.0 corresponding to the ethyl group. In addition, the data demonstrate that the hydrazone product exists as a 1:2 equilibrium mixture of *syn*- (**3**) and *anti*-forms (**4**) (Scheme 1). The major isomer is assumed to have the *syn*-stereochemistry **4** based on the expectation that its NH proton would be deshielded in the ^1^H-NMR spectrum as a result of potential hydrogen bonding with the carbonyl ester moiety (*cf.* Scheme 1). 

Brecknell *et al.* [18] was able to isolate and characterize both isomers. The predominance of the anti-form of2-phenylhydrazonoacetate was attributed to stereoelectronic effects. Recently Al-Awadi *et al.* have shown that in similar systems stereoelectronic effects are more important than hydrogen bonding in governing syn-anti ratios [19]. 

**Scheme 2:**
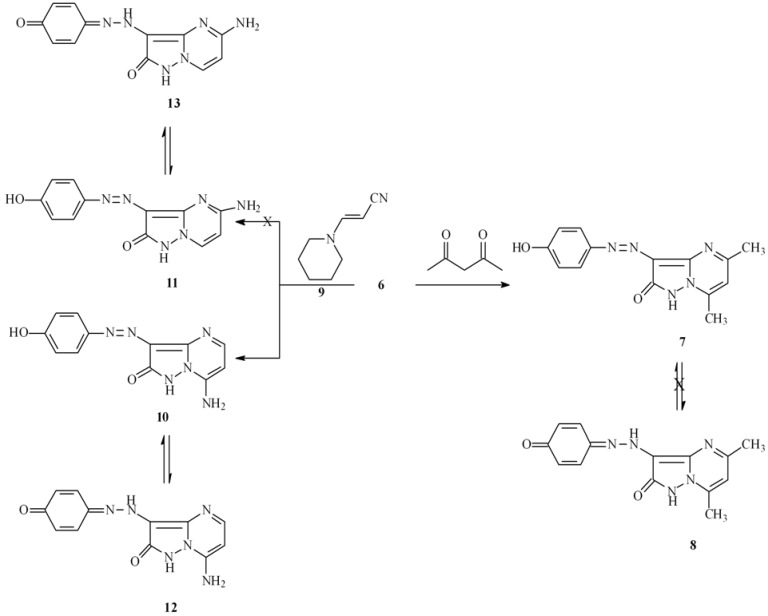
Synthesis of 3-(4-hydroxyphenylazo)-5,7-dimethylpyrazolo[1,5-*a*]pyrimidin-2-one(**8**) and 7-amino-3-(4-hydroxyphenylazo)-pyrazolo[1,5-a]pyrimidin-2-one (**12**).

Hydrazone **3** undergoes a smooth reaction with hydrazine hydrate to yield 4-(4-hydroxyphenyl-hydrazonopyrazolone **6**. The structure and the chemistry of such compounds have received considerable attention [20,21,22]. We have observed that **6** readily condenses with acetylacetone to yield the pyrazolo[1,5-a]pyrimidine **7**, which has the potential of existing in tautomeric form **8**. However, the 2D ^1^H-NMR analysis results indicate that it exists in only one form having the nonquinoidal structure **7 **(*cf.* Scheme 2).

**Figure 1:**
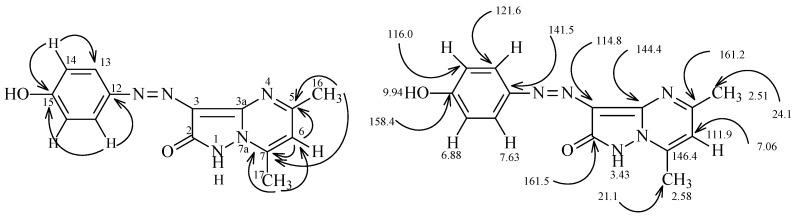
^1^H- and ^13^C-NMR spectroscopy assignments of compound **7**.

The important features of the HMBC 2-D ^1^H, and ^13^C signals are shown in Figure 1. H^6^ at 7.06 ppm correlates with C^5^ and C^7^ at 161.2 and 146.4 ppm; H^13^ at 7.63 ppm correlates with C^12^ and C^15^ at 141.5 and 158.4 ppm; H^14^ at 6.88 ppm correlates with C^13^ and C^15^ at 121.6 and 158.4 ppm; H^16^ at 2.51 ppm correlates with C^5^ and C^7^ at 161.2 and 146.4 ppm; and H^17^ at 2.58 ppm correlates with C^6^ and C^7^ at 111.9 and 146.4 ppm. 

^1^H-^15^N HMBC experiments add further support to the assignment of structure **7**, such as the chemical shifts observed for N^7a^ at 210 ppm, N^4^ at 260 ppm and N^12a^ at 370 ppm, the cross peak correlations for coupling of H^6^ at 7.06 ppm is also observed with N^7a^ at 210 ppm (*^3^J*) (H^6^, N^7a^), and N^4^ at 260 ppm (*^3^J*) (H^6^, N^4^). Interestingly, coupling of H^16^ at 2.51 ppm with only N^4^ at 260 ppm (*^3^J*) (H^16^, N^4^) is observed, coupling of H^17^ at 2.58 ppm takes place only with N^7a^ at 210 ppm (*^3^J*) (H^17^, N^7a^) (Figure 1).

**Figure 2:**
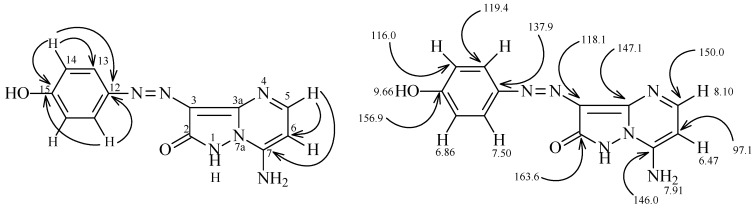
^1^H and ^13^C NMR spectroscopy assignments of compound **10**.

Hydroxyphenylhydrazonopyrazolone **6** also reacts with 2-piperidinylacrylonitrile (**9**) to yield a product for which structures **10**- **13** are possible based on the absence of an amino-proton resonance in their ^1^H-NMR spectra and a cyano band in the IR spectrum. The assignment of structure **10** was made by H-C correlations in the HMBC 2-D experiments (Figure 2).

The correlations of H^5^ at 8.10 ppm with C^6^ and C^7^ at 97.1 and 146.0 ppm, H^13^ at 7.51 ppm with C^12^ and C^15^ at 137.9 and 156.9 ppm, and H^14^ at 6.90 with C^12^, C^13^ and C^15^ at 137.9, 119.4 and 156.9 ppm were observed, further structural information came from the results of^ 1^H-^15^N HMBC experiments, the chemical shifts for N^7a^ and N^4^ are 198.7 and 234.2 ppm, respectively, cross peak correlations for coupling of the shielded proton H^6^ at 6.47 ppm takes place with N^7a^ at 198.7 ppm (*^3^J*) (H-6, N-7a) and N^4^ at 234.2 ppm (*^3^J*) (H-6, N-4), coupling of the deshielded proton H^5^ at 8.10 ppm with only N^4^ at 234.2 ppm (*^3^J*) (H-5, N-4) is also observed.

**Scheme 3:**
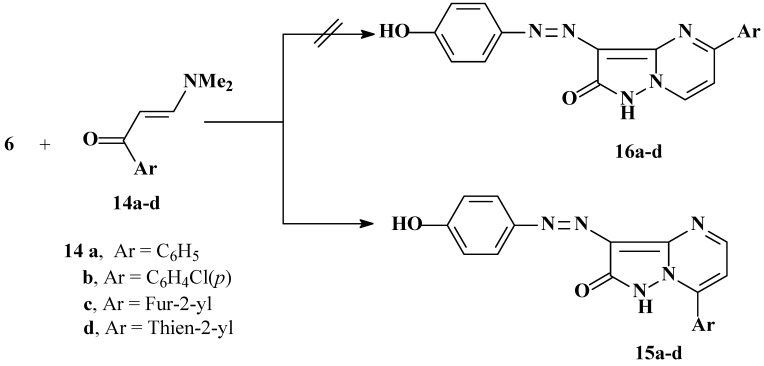
Synthesis of pyrazolo[1,5-a]pyrimidinone derivatives.

**Figure 3:**
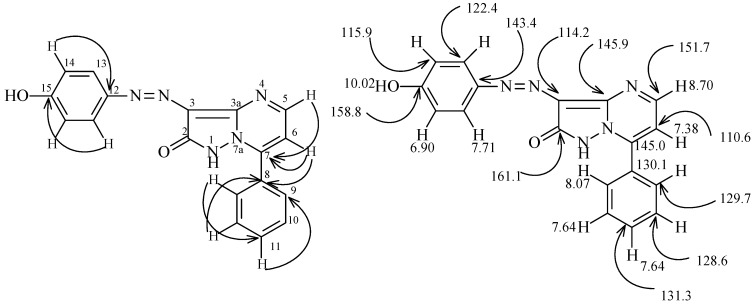
^1^H- and ^13^C-NMR of compound **15a**.

Compound **6** reacts with enaminones **14a-d** to yield the corresponding pyrazolo[1,5-a]pyrimidines **15a-d **(*cf.* Scheme 3) whose identities were established by using 2D NMR experiments (Figure 3).

^1^H and ^13^C signal assignments and HMBC 2-D derived H-C correlations for **15a**. The important features of the HMBC correlations are the H^5^ at 8.7 ppm with C^7^ at 145.0 ppm, H^6^ at 7.38 ppm with C^7^ and C^8^ at 145.0 and 130.1 ppm, H^9^ at 8.07 ppm with C^11^ at 131.3 ppm, H^10^ at 7.64 ppm with C^8^ at 130.1 pm, H^11^ at 7.64 ppm with C^9^ at 129.7 ppm, H^13^ at 7.71 ppm with C^15^ at 158.8 ppm, and H^14^ at 6.90 ppm with C^12^ at 143.4 ppm.

The possible formation of regioisomers **16** in reactions of **6** with enaminones **14a-d** is ruled out based on ^1^H-^15^N HMBC experimental results. Conclusive data for the structure of **15a** include chemical shifts for N^7a^ at 208.7 ppm and N^4^ at 270.4 ppm. Cross peak correlations for coupling of the shielded proton H^6^ at 7.39 ppm are observed with N^7a^ at 208.7 ppm (*^3^J*) (H^6^, N^7a^) and N^4^ at 270.4 ppm (*^3^J*) (H^6^, N^4^). Coupling of the deshielded proton H^5^ at 7.70 ppm with N^4^ at 270.4 ppm (*^3^J*) (H^5^, N^4^) only is also observed. The results demonstrate that the phenyl moiety is located at C^7^ and not at C^5^.

### 2.2. Dyeing and Fastness Properties

Arylazopyrazolopyrimidine derivatives **6**, **7** and **15a-d** were tested as dyes for polyester fibers using the high temperature dyeing method (HT) by employing microwave heating as an energy source. The physical and analytical data for the dyed fibers, given in Table 1 and Table 2, reflect the efficiency of the microwave irradiation, leading to a large increase in dye uptake and dyeing rate along with a performance of dye leveling and color homogeneity.

#### 2.2.1. Color strength

Data in Table 1 reveals that the color strengths (K/S) of dyed polyester fabrics are directly proportional to the amounts of the dyes applied (% *o.m.f*.). The hues of the fabrics treated with the azo dyes were found to vary from yellowish-orange to dark orange, based on the substitution patterns. Differences in the color strength observed depend on the substitution patterns, ‘auxochromes’, present in the arylazopyrazolopyrimidine disperse dyes referred to as ‘chromogens’ [14,23,24]. Data in Table 1 clearly shows that the magnitude of color strength obtained using dye **15d** is much larger than that for **15c**.

**Table 1:** Color strength of dyed polyester using high temperature dyeing method.

Dye	Color shade on polyester	Color strength (K/S) % Dye o.m.f.			
		1	2	3	4
**6**	Yellowish-orange	1.17	2.12	2.47	2.49
**7**	Pale orange	2.19	3.79	4.09	6.96
**15a**	Dark orange	5.66	5.95	7.12	8.31
**15b**	Dark orange	5.43	5.81	7.91	10.04
**15c**	Orange	4.40	4.64	4.85	6.01
**15d**	Orange	4.08	4.73	6.02	7.06

#### 2.2.2. Wash fastness

Fastness data, obtained by measuring color intensity changes in the dyed fabrics, are given in Table 2. It shows that wash fastness varies with the substitution patterns in the dyes, where an increase occurs when stronger electron-attracting groups are present on the aryl moieties. This might be attributed to the fact that these disperse dyes are mainly dispersed within the pores in the polyester fabrics and are held in their places by different forces including Van der Waal forces. Electron-attracting groups enable stronger Van der Waal forces and also hydrogen bonding with the dyed fabrics that increases their stability to washing. 

#### 2.2.3. Light fastness

The light fastness properties of substituted of fabrics treated with the disperse dyes were determined (Table 2). The low light fastness properties observed are most likely result the photochemical reactivity of the arylazopyrazolopyrimidine dyes. Indeed, the results of LCMS monitoring of the photolysate, obtained by irradiation of 3-(4-hydroxyphenylazo)-7-phenylpyrazolo[1,5-a]pyrimidin-2-one (**14a**), under 16 W low pressure mercurey arc-lamp (254 nm) in acetonitrile for 48 h, supported this finding. Elnagdi *et al.* [25] observed that 3,4-diarylaminopyrazoles are reactive when treated under reflux with AcOH-H_2_SO_4_ mixture. 

**Table 2:** Fastness properties of azo disperse dyes on polyester fabrics, prepared by using high temperature dyeing method*.*

Dye	Wash fastness *^a,b^*			Light fastness
	Alt	SC	SW	
**(1% o.m.f. dyeing)**				
**6**	5	5	5	2-3
**7**	5	5	5	2
**15a**	5	5	5	2-3
**15b**	5	5	5	3
**15c**	4	5	4	2-3
**15d**	5	5	5	2
**(2% o.m.f. dyeing)**				
**6**	5	5	5	2-3
**7**	5	5	5	2
**15a**	5	5	5	2
**15b**	5	5	5	2-3
**15c**	4	5	4	2-3
**15d**	5	5	5	2
**(3% o.m.f. dyeing)**				
**6**	5	5	5	2-3
**7**	5	5	5	2
**15a**	4-5	5	4-5	2
**15b**	4-5	5	4-5	2
**15c**	4	5	4	2-3
**15d**	4-5	4-5	4-5	2
**(4% o.m.f. dyeing)**				
**6**	5	5	5	2-3
**7**	5	5	5	2
**15a**	5	5	5	2
**15b**	5	5	5	2
**15c**	4	5	4	2
**15d**	4-5	5	4-5	2

*^a^* ISO CO2/CO41; *^b^* Alt = alteration; SC = staining on cotton; SW = staining on wool.

## 3. Experimental

### 3.1. General

All melting points were recorded on a Gallenkamp apparatus and are uncorrected. IR spectra were recorded in KBr disks on a Perkin Elmer System 2000 FT-IR spectrophotometer. ^1^H- and ^13^C-NMR spectra were recorded on a Bruker DPX 400 MHz super-conducting NMR spectrometer. Mass spectra were measured on a VG Auto-spec-Q instrument (high resolution, high performance, tri-sector GC/MS/MS) and by LC-MS using an Agilent 1100 series LC/MSD with API-ES/APCI ionization mode. Microanalyses were performed on a LECO CH NS-932 Elemental Analyzer. The microwave oven used is a single mode cavity Explorer Microwave (CEM Corporation, Matthews, NC, USA) and irradiate in heavy-walled Pyrex tube (capacity 10 mL and 80 mL for dyeing). The color strengths (K/S) of the dyed polyester fabrics and the color fastness to light were evaluated at the Dyeing, Printing and Textile Auxiliaries Department, Textile Research Division, National Research Centre, Giza, Egypt.

### 3.2. General Procedure for the Synthesis of azo Disperse Dyes

*Ethyl cyano(4-hydroxyphenylhydrazono)acetate* (**3**). *p*-Aminophenol (10.9 g, 0.1 M) was dissolved in concentrated HCl (30 m) and water cooled in ice (20 mL) and then NaNO_2_ (7 g) in water (50 mL) was added in portions. A mixture of ethyl cyanoacetate (10 g, 0.1 M), NaOAc (20 g), ethanol (15 mL) and water (50 mL) was prepared separately and cooled in ice. The diazonium salt solution was added slowly to the second solution, with ice cooling. The cooled mixture was stirred for 0.5 h and filtered to give brown crystals, which were crystallized from alcohol/water to yield 2.0 g (86%) of **3**, m.p. 273–274 °C. MS: *m/z* = 331 (M^+^, 100%), 238 (50%), 182 (20%). IR: 3432, 3066, 3003, 1623, 1591, 1531, 1461, 1232, 829 cm^–1^. ^1^H-NMR (DMSO-d_6_) 13.02 (s, 1H, NH, *syn*), 12.16 (s, 1H, NH, *anti*), 9.68 (s, 1H, OH, *syn*), 9.57 (s, 1H, OH, *anti*), 7.36 (d, 2H, *J* = 7.4 Hz, *syn*), 7.31 (d, 2H, *J* = 8.0 Hz, *anti*), 6.81 (d, 4H, *J* = 8.0 Hz, *syn*+*anti*), 4.30 (q, 2H, *syn*), 4.24 (q, 2H, *anti*), 1.32 (t, 3H, *syn*), 1.28 ppm (t, 3H, *anti*). ^13^C-NMR (DMSO-d_6_) 161.4, 161.3, 155.9, 155.3, 134.2, 133.5, 118.0, 117.9, 116.4, 116.0, 115.9, 112.0, 101.9, 101.4, 61.7, 61.2, 14.2, 14.0 ppm. HRMS = 233.0794, requires C_11_H_11_N_3_O_3_ 233.0794.

*5-Amino-4-[(4-hydroxyphenyl)-hydrazono]-2,4-dihydropyrazol-3-one* (**6**). A mixture of **3** (2.33 g, 10 mmol), hydrazine hydrate (2.5 mL) in ethanol (20 mol) was stirred at reflux for 3-4 h. The solvent was removed under vacuum and the formed solid was collected and crystallized from ethanol/water to give **6**. Red brown crystals from alcohol, yield 2.6 g (73%), m.p. 263 °C. MS: *m/z* = 219 (M^+^, 100%), 126 (10%), 108 (60%). IR: 3472, 3361, 3283, 3165, 3130, 1678, 1626, 1268, 820 cm^–1^. ^1^H-NMR (DMSO-d_6_) 12.90 (br, 1H, NH), 10.45 (s, 1H, NH), 9.50 (br, 1H, OH), 7.37 (d, 2H, *J* = 8.0 Hz) 6.78 (d, 2H, *J* = 8.0 Hz) 5.71 ppm (br, 2H, NH_2_); ^13^C-NMR (DMSO-d_6_) 160.7, 158.8, 151.6, 144.9, 143.4, 131.3, 130.0, 129.6, 128.5, 122.4, 115.9, 114.1, 110.6, 115.7 ppm.

#### General procedure for the synthesis of pyrazolo[1,5-a]pyrimidines **7**, **10** and **15a-d**

A mixture containing **6 **(0.22 g, 10 mmol), and acetylacetone, 2-piperidinylacrylonitrile or enaminones **5a-d** (1 mmol) in acetic acid (5 mmol) was irradiated in a microwave oven at 140 °C for 2 min. The mixture was then poured into ice water (50 mL). The formed was collected and crystallized from the appropriate solvent (see below).

*3-(4-Hydroxyphenylazo)-5,7-dimethylpyrazolo[1,5-a]pyrimidin-2-one* (**7**). Red crystals from DMF, yield 2.79 g (73%), m.p. 277-278 °C. MS: *m/z* = 283 (M^+^, 100%), 190 (90%), 162 (30%). IR: 3458, 3113, 3013, 1620, 1534, 1423, 1250, 1139, 840 cm^–1^. ^1^H-NMR (DMSO-d_6_) 9.94 (s, 1H, OH), 7.63 (dd, 2H, H^13^*J =* 7.4, 1.8 Hz), 7.06 (s, 1H, H^6^), 6.88 (dd, 2H, H^14^, *J* = 7.4, 1.8 Hz), 3.43 (br, 1H, NH), 2.58 (s, 3H), 2.51 ppm (s, 3H). ^13^C-NMR (DMSO-d_6_) 161.5, 161.2, 158.4, 146.4, 144.4, 141.5, 121.6, 116.0, 114.8, 111.9, 24.0, 16.5 ppm. Anal. Calcd. for C_14_H_13_N_5_O_2_ (283.3): C 59.36; H 4.63, N 24.72. Found: C 59.30; H 4.57; N 24.71. 

*7-Amino-3-(4-hydroxyphenylazo)pyrazolo[1,5-a]pyrimidin-2-one* (**10**). Red crystals from DMF, yield 2.79 g (73%), m.p. 273 °C. MS: *m/z* = 270 (M^+^, 75%), 177 (35%), 150 (100%). IR: 3353, 3146, 2926, 1649, 1620, 1543, 1444, 1239, 1176, 823 cm^–1^. ^1^H-NMR (DMSO-d_6_) 9.66 (s, 1H, OH), 8.10 (d, 1H, H^5^, *J =* 6.0 Hz), 7.91 (br, 2H, NH_2_), 7.50 (d, 2H, *J =* 8.0 Hz), 6.86 (d, 2H, *J* = 8.0 Hz), 6.47 ppm (d, 1H, H^6^, *J* = 6.0 Hz). ^13^C-NMR (DMSO-d_6_) δ 163.6, 156.9, 150.0, 147.1, 164.0, 137.9, 119.6, 118.1., 116.0, 97.1 ppm. Anal. Calcd. for C_12_H_9_N_6_O_2_ (269.2): C 53.53; H 3.37, N 31.21. Found: C 53.47; H 3.27; N 31.05. 

3*-(4-Hydroxyphenylazo)-7-phenylpyrazolo[1,5-a]pyrimidin-2-one* (**15a**). Red crystals from DMF, yield 2.6 g (78%), m.p. 273-274 °C. MS: m/z = 331 (M+, 100%), 238 (50%), 182 (20%). IR: 3432, 3066, 3003, 1623, 1591, 1531, 1461, 1232, 829 cm^–1^. ^1^H-NMR (DMSO-d_6_) 10.02 (s, 1H, OH), 8.70 (d, 1H, H^5^, *J* = 4.2 Hz), 8.07 (dd, 2H, H^9^, *J* = 7.2, 1.8 Hz), 7.71 (dd, 2H, H^13^, *J* = 7.2, 1.8 Hz), 7.64 (m, 3H, H^10^, H^11^), 7.38 (d, 1H, H^6^, *J* = 4.2 Hz), 6.90 (dd, 2H, H^14^, *J* = 7.2, 1.8 Hz), 3.36 ppm (br, 1H, NH). ^13^C-NMR (DMSO-d_6_) 161.1, 158.8, 151.7, 145.0, 145.9, 143.4, 131.3, 130.1, 129.7, 128.6, 122.4, 115.9, 114.2, 110.6 ppm. Anal. Calcd. for C_18_H_13_N_5_O_2_ (331.3): C 65.25; H 3.95; N 21.14. Found: C 64.93; H 3.92; N 21.03.

*7-(4-Chlorophenyl)-3-(4-hydroxyphenylazo)pyrazolo[1,5-a]pyrimidin-2-one* (**15b**). Red crystals from DMF, yield 2.7 g (84%), m.p. 285–286 °C. MS: *m/z* = 365 (M^+^, 100%), 272 (60%), 149 (25%). IR: 3430, 3227, 3095, 1633, 1596, 1514, 1483, 1445, 1253, 821 cm^–1^. ^1^H-NMR (DMSO-d_6_) 10.02 (s, 1H, OH), 8.71 (d, 1H, *J* = 4.8 Hz), 8.13 (dd, 2H, *J* = 7.2, 1.8 Hz), 7.71 (m, 4H), 7.42 (d, 1H, *J* = 4.8 Hz), 6.89 (dd, 2H, *J* = 7.2, 1.8 Hz), 3.38 ppm (br, 1H, NH). ^13^C-NMR (DMSO-d_6_) 161.0, 158.8, 151.6, 144.9, 144.7, 143.3, 136.1, 131.5, 128.8, 128.6, 122.4, 115.9, 114.2, 110 ppm. Anal. Calcd. for C_18_H_12_ClN_5_O_2_ (365.8): C 59.11; H 3.31, N 19.15. Found: C 58.78; H 3.32; N 18.90.

*7-Furan-2-yl-3-(4-hydroxyphenylazo)pyrazolo[1,5-a]pyrimidin-2-one* (**15c**). Red crystals from DMF, yield 2.7 g (84%), m.p. 291–292 °C. MS: m/z = 321 (M^+^, 100%), 228 (45%), 172 (15%). IR: 3429, 3109, 3035, 1626, 1594, 1536, 1462, 1238, 1132, 828 cm^–1^. ^1^H NMR (DMSO-d_6_) 10.01 (s, 1H, OH), 8.67 (d, 1H, *J* = 4.8 Hz), 8.23 (d, 1H, *J* = 3.6 Hz), 8.15 (d, 1H, *J* = 3.6 Hz), 7.69 (d, 2H, *J* = 8.4 Hz), 7.62 (d, 1H, *J* = 4.8 Hz), 6.95 (m, 1H), 6.89 (d, 2H, *J* = 8.4 Hz), 3.37 ppm (br, 1H, NH). ^13^C-NMR (DMSO-d_6_) 162.1, 158.6, 150.5, 147.9, 145.0, 142.6, 141.9, 134.6, 121.9, 120.9, 115.9, 114.7, 113.6, 105.8 ppm. Anal. Calcd. for C_16_H_11_N_5_O_3_ (321.3): C 59.81; H 3.45, N 21.80. Found: C 59.78; H 3.34; N 21.81. 

*3-(4-Hydroxyphenylazo)-7-thiophen-2-yl-pyrazolo[1,5-a]pyrimidin-2-one* (**15d**). Red crystals from DMF, yield 2.6 g (77%), m.p. 284–285 °C. MS: m/z = 337 (M^+^, 100%), 244 (50%), 188 (20%). IR: 3429, 3104, 3035, 1623, 1592, 1535, 1462, 1238, 1160, 826 cm^–1^. ^1^H-NMR (DMSO-d_6_) 9.99 (s, 1H, OH), 8.62 (d, 1H, *J* = 4.8 Hz), 8.55 (d, 1H, *J* = 3.6 Hz), 8.19 (d, 1H, *J* = 4.8 Hz), 7.98 (d, 1H, *J* = 4.2 Hz), 7.67 (d, 2H, *J* = 8.4 Hz), 7.41 (t, 1H, *J* = 4.2 Hz), 6.89 (d, 2H, *J* = 8.4 Hz), 3.38 ppm (br, 1H, NH). ^13^C-NMR (DMSO-d_6_) 162.6, 158.2, 150.1, 145.5, 140.4, 139.1, 136.2, 133.1, 129.4, 127.8, 121.3, 116.0, 115.7, 108.0 ppm. Anal. Calcd. for C_16_H_11_N_5_O_2_S (337.4): C 56.96; H 3.29, N 20.76 ; S 9.50. Found: C 56.88; H 3.30; N 20.71 ; S 9.46.

### 3.3. High Temperature Dyeing Method (HT)

#### 3.3.1. Materials

Scoured and bleached polyester 100% (150 130 g/m^2^, 70/2 denier) was obtained from El-Shourbagy Co., Egypt. The fabric was treated before dyeing with a solution containing non-ionic detergent (Hostapal CV, Clariant-Egypt, 5 g/L) and sodium carbonate (2 g/L) in a ratio of 50:1 at 60 °C for 30 min, then thoroughly washed with water and air dried at room temperature.

#### 3.3.2. Dyeing

Dyeing of polyester fabrics was carried out at 130 °C for 60 min, under pressure in a microwave oven in a 20:1 liquor ratio and pH 5.5 in the presence of a 1:1 ratio of the dispersing agent sodium lignin sulphonate and the with a 1–4% shade. After dyeing, the fabrics were thoroughly washed and then subjected to a surface reduction cleaning [(5 g NaOH + 6 g sodium hydrosulphite)/L]. The samples were heated in this solution for 10 min. at 60 °C and then thoroughly washed and air-dried.

### 3.4. Color Measurements and Analyses

#### 3.4.1. Color measurements of the dyed fabrics

The color yields of the dyed samples were determined by using the light reflectance technique performed on a Perkin-Elmer (Lambda 3B) UV/VIS Spectrophotometer. The color strengths, expressed as K/S values, were determined by applying the Kubelka-Mink equation as follows:

K/S = [(1–R)^ 2^ / 2R] – [(1–R_o_)^ 2^ / 2R_o_]

where *R* = decimal fraction of the reflectance of the dyed fabric; *R_o_* = decimal fraction of the reflectance of the undyed fabric; *K* = absorption coefficient; *S* = scattering coefficient.

#### 3.4.2. Fastness testing

After washing using 2 g/L of the non-ionic detergent Hostapal CV at 80 °C for 15 min, the dyed fabrics were tested, employing ISO standard methods [26]. Wash fastness tests were carried out in accordance with ISO 105-C04 (1989), in which 5 g/L soap and 2 g/L soda ash solution were used at 95 °C for 30 min in the presence of 10 steel balls (liquor ratio 50:1) and color fastness to light (carbon arc), ISO 105-B02 (1988). 

## 4. Conclusions

In summary, a series of novel monoazo disperse pyrazolopyrimidine dyes were synthesized via a sequence involving initial coupling of ethyl cyanoacetate with 4-hydroxybenzenediazonium chlorides. Subsequent treatment with hydrazine hydrate gave the corresponding pyrazolohydrazone that was then treated with either 2,5-pentandione or arylenaminoketones to give the target pyrazolo[1,5-a]pyrimidine dyes. The dyes produced in this manner were then applied to polyester fibers by using HT dyeing conditions and microwave heating. The dyed fabrics, which displayed yellow to yellow brown hues on polyester fibers, have low fastness levels to light and excellent wash fastness.
